# Letter from M. Vernois

**Published:** 1840-06-20

**Authors:** 


### FOREIGN CORRESPONDENCE.

### LETTER FROM M. VERNOIS.

### No. III.

*The Late Concours for the Chair of Internal Pathology in the School of Medicine of Paris.*

Paris, 27th April, 1840.

To the Editors of the Medical Examiner.

The month which has just elapsed has witnessed an event of considerable importance in the Medical School of Paris. A titular professor of internal pathology has been named at the close of a public *concours*, which had been prolonged for four months. Few things excite more lively interest in the capitol than a *concours* for one of the chairs of medicine. The number of the competitors, their relative ability and influence, their connections both with society and with the instructing body actually in power, which are so many isolated motives, so many different elements, that each one brings into play according to his energy and capability,—all contribute to form a subject of great interest, and agitate not a little the medical world. In fact, the result of this solemn contest is fraught with many important consequences: it places in one of the best chairs of the whole range of medical education, the competitor judged most worthy to transmit and develop the immense scientific budget designated under the name of pathology; it opens to the conqueror a share in the reputation justly enjoyed by our faculty in neighboring nations; to say nothing of a point which some of our great men duly appreciate—the comfortable pecuniary as well as scientific position which the successful candidate attains. Never at any other period were the competitors more at their ease, nor did they enjoy a more complete freedom of opinion and of doctrine. The school, to enter which was the object of their ambition, can no longer be characterized by the unity of its instruction, the uniformity of its principles, and harmony of its doctrines. Every member of it now looks at science after his own fashion. The Hôtel Dieu is the antipodes of la Charité, and wide are the limits which separate the clinique of the faculty from the chair of general pathology. While the School of Montpelier is endeavouring to revive its ancient devise, so long obscured by the eclat of the pathologo-anatomical glare of Paris; while the School of Strasburg remains faithful to its traditions of pure anatomy, and avails itself, through its neighbourhood to Germany, of the researches in physiology, natural philosophy, and medical literature, cultivated in that country, our faculty, our School of Paris is every day separating more and more its bundle of reeds, opening its doors to every wind of doctrine, and becoming the representative, not so much of a distinct body, as an assemblage of every shade of opinion known in scientific Europe. This state of things has its advantages as well as its inconveniences. It was, therefore, interesting to study in a public *concours*, from the character of the efforts, of the questions assigned, and the manner in which they were solved, both the spirit of the judges and the competitors. As regards medical pathology, it was, so to say, to collect up to a certain point the contemporaneous history of the progress, the developement, and the tendencies of this branch of the art. From the details into which we are about to enter, and the exact enumeration which will be given of the circumstances attending this *concours*, your readers will be enabled to judge, better certainly than by mere description, what is just now the School of Paris, its capacities, and its prospects.

*Judges of the Concours:*—From the Professors, MM. Andral, Fouquier, Chomel, Duméril, Trousseau, Cruveilhier, Paul Dubois, Gerdy—Supplementary, MM. Roux, Marjolin. —From the Academy of Medicine: MM. Roche, Bally, Honoré, Rayer—Supplementary, M. Bricheteau.

*Competitors:*—MM. N. Guillot, Dalmas, Legroux, Requin, Hourmann, Cazenave, Piorry, C. Broussais, Gilbert, Dubois d'Amiens, Gendrin, and Combette.

*Requisitions:*—1. A written composition; the same for each competitor. 2. A lecture of an hour's length, after twenty-four hours' preparation, upon a subject selected by lot. 3. A lecture of an hour's length, the same for two candidates, after three hours' preparation, upon a subject likewise selected by lot. 4. A printed thesis. 5. A discussion of this thesis, successively by four competitors. 6. Examination of the anterior claims of each candidate.

Were we to look at the *concours*, and the means by which it is carried into execution, simply as they profess to be themselves, and divested of every manœuvre and dishonourable intrigue, we should certainly find in the conditions proposed every guarantee for the attainment of the desired object. The candidate is thus enabled to bring forward and display his erudition, style, and science, in the written composition; his judgment, logic, medical spirit, and elocution, in the oral efforts; his views of pathology, his facility of disposing of his material, arranging facts, and deducing conclusions, in his thesis; finally, as regards his anterior claims, from his past character, his future usefulness and success might in a great measure be anticipated. But we live in an age in which the most sacred things have been blighted, and the noblest institutions diverted from their end. Hence we need not be surprised to find, that in the progress of the result of this *concours*, human passions force their way in to play their accustomed part, and that in the struggle for the prize, elements of competition are brought to bear, altogether perverting the original wise intentions of legislation.

Without predilection or bias in any direction, I have been an attentive observer of the contest, and propose to offer a rapid analysis of it to your readers. The question presented for written composition was, "*On Fever.*"—Undoubtedly, few subjects could have offered so many difficulties in solution; but, on the other hand, few could afford scope for so much developement, or so many important considerations, including, so to say, the entire history of the progress and vicissitudes of medicine. The different epochs of the science all offered themselves for review, with their various systems, modified with the successive experience of ages, until we came to consider fever as something else than a vague word without positive definition. Again, was to be shown the actual point of connection between existing opinions and the views which have prevailed in medicine, from the time of Hippocrates to our own. From these views were to be deduced positive conclusions, if based upon facts, or the expression of doubts, if such were the legitimate result of such a discussion of the premises. Here, then, was an ample field opened to the candidates for the display of solid erudition, accurate powers of analysis, just appreciation of the merits of the past and present, with knowledge of fever derived from the bedside of the patient, considering it as the basis of the other phenomena of disease,—such, I say, was the field opened on a subject which, in the hands of Pinel and Broussais, has in our days shaken the medical world to its very centre. The liberality of the judges was shown in the selection of a question so very general; and amply did the competitors avail themselves of this liberality. From this first essay, it was not difficult to seize the aspect of the *concours*, to classify the combatants, and see under what banners they fought. Thus, MM. Legroux, Guyot, Cazenave, C. Broussais, Dalmas, became the champions of the anatomical school, liberally understood; M. Gendrin showed himself a pure anatomo-pathologist, and gave an analysis of the researches published by him on this subject. And M. Gibert, alone, raised the standard of Hippocratic medicine, and, it must be confessed, acquitted himself very skilfully of this difficult task. The other competitors did not travel out of the common path; and from their first steps, occupied but an insignificant space in the arena.

The second trial consisted in the delivery of a lecture an hour long, upon a subject selected but a day previous. This trial is unquestionably one of the most important. Every one has a fair opportunity of preparing himself; all the sources of erudition are open to him; and what he shows himself to-day, he will probably be for the future. Here all his qualities unfold themselves; the bent of his mind, the character of his judgment, his talent for exposition, for eloquence; his delivery, his gestures—are all brought before the public eye. As if he were already professor, he delivers from his chair a lecture, for the preparation of which he has had ample time: from the history of the science he has been able to *select* whatever he deemed of capital importance; he has had leisure to *classify* his ideas, so as develope them with arrangement and clearness; in a picture of just limits, he may have arranged his colours in harmonious lines and fair proportions; finally, if he be endowed with a voice of eloquence and persuasion, he may at once captivate and arrest the attention of his auditory. It was, then, important to display, in this trial, not a fatiguing erudition, and too spiritual a phraseology; for erudition in this case was at the command of all, and wit is not science, in medicine. Unfortunately, among the candidates were found some men most unluckily erudite, and some would-be wits. By the rest, the trial was in general but feebly sustained. And yet the judges had been prodigal of noble and great subjects. Your readers will be able to perceive this from the following enumerations: The candidates were called upon successively to discuss,—pus and suppuration; the sympathetic phenomena of diseases; rheumatism and gout; paralysis; convulsions; nervous affections; eruptive fevers in general; tubercles; puerperal fevers; delirium; vomiting; and, lastly, the entozoaria. Certainly, the judges deserved well of science, in thus proposing a series of questions, all of about the same importance, and all so put as to be susceptible of being treated in a general manner. I have said that the trial was but feebly sustained. In fact, among the candidates there were no minds of high generalizing powers; no minds that could seize with clearness all the bearings of a subject, so as afterwards to present a rapid and intelligent analysis of it. There are in medicine many minds of descriptive powers; in it, as in natural history, description has its advantages and its degree of merit; but it is very inferior to that high power of synthesis, which looks from high upon facts scattered and without limits, and collects them together, weighs them, compares them, and from apt collocation of them, deduces those general laws which become the expression of all the great truths of science. We must repeat, that but few of the candidates understood the extent of the requisitions upon them, and the just importance of the task assigned them. We may, however, mention the lectures of MM. Gendrin, upon *paralysis;* Dalmas upon the *entozoaria*, and Legroux upon eruptive fevers. MM. Piorry, upon *tubercles*, and Gibert, upon *nervous affections*, showed their knowledge of the qualifications necessary in a professor of pathology.

(*To be concluded in our next.*)

